# Tako-tsubo Syndrome as First Manifestation in a Case of Pheochromocytoma Developed From a Non-functional Adrenal Incidentaloma

**DOI:** 10.3389/fendo.2020.00051

**Published:** 2020-02-14

**Authors:** Pierpaolo Falcetta, Francesca Orsolini, Eleonora Molinaro, Paolo Vitti, Massimo Tonacchera

**Affiliations:** Section of Endocrinology, Department of Clinical and Experimental Medicine, University Hospital of Pisa, Pisa, Italy

**Keywords:** adrenal incidentaloma, pheochromocytoma, tako-tsubo syndrome, catecholamine, infarction, ventricular dysfunction

## Abstract

**Background:** Pheochromocytoma is a catecholamine secreting tumor that, in extremely rare cases, may develop over time from a non-functional adrenal adenoma. Catecholamine excess can lead to a kind of cardiomyopathy similar to that seen in tako-tsubo syndrome (TTS).

**Case report:** A 69 years old female with a history of type 2 diabetes, hypertension, and a non-functional right adrenal adenoma diagnosed 3 years earlier was referred to our center for further investigations. During the evaluation, she had a hypertensive crisis with chest pain, tachycardia, and diaphoresis. Suspecting an acute coronary syndrome, she underwent coronary angiography, which showed the typical features of TTS. The high 24 h-urinary metanephrines excretion and abdominal MRI findings were suggestive of pheochromocytoma. Right laparoscopic adrenalectomy was performed, with the resolution of all symptoms. Pathology findings confirmed the diagnosis of pheochromocytoma. After 12 months, the patient was still asymptomatic, with the echocardiography displaying a complete recovery of the left-ventricular function.

**Conclusions:** The development of a pheochromocytoma from an adrenal non functional adenoma is an extremely rare event, but potentially life-threating because of the catecholamine-associated cardiovascular toxicity. In particular, TTS is a form of cardiomyopathy that has been increasingly described as associated with catecholamine-secreting tumors. The exclusion of pheochromocytoma in a patient with TTS has important therapeutic implications, since the administration of β-blockers may be extremely harmful in patients with catecholamine surge in the absence of adequate α-blockage.

## Introduction

An adrenal incidentaloma (AI) is an adrenal mass unexpectedly found during an imaging examination performed for reasons other than a suspected adrenal disease. In most cases, adrenal masses are non-functional at the time of diagnosis and don't develop hormone secretion over time. Still, a minority (~5%) have proved to be a pheochromocytoma, a rare neuroendocrine tumor producing catecholamines, originating from chromaffin cells of the adrenal medulla. When the tumor arises from extra-adrenal paraganglia is called paraganglioma (PPGL) (1). Cardiac involvement in pheochromocytoma/PPGL is frequent, and, in some cases, it includes a transient form of cardiomyopathy very similar to that seen in tako-tsubo syndrome (TTS). We reported the case of a patient with TTS caused by a pheochromocytoma evolved from a long-standing non-functional AI.

## Case Presentation

**Figure 1:**
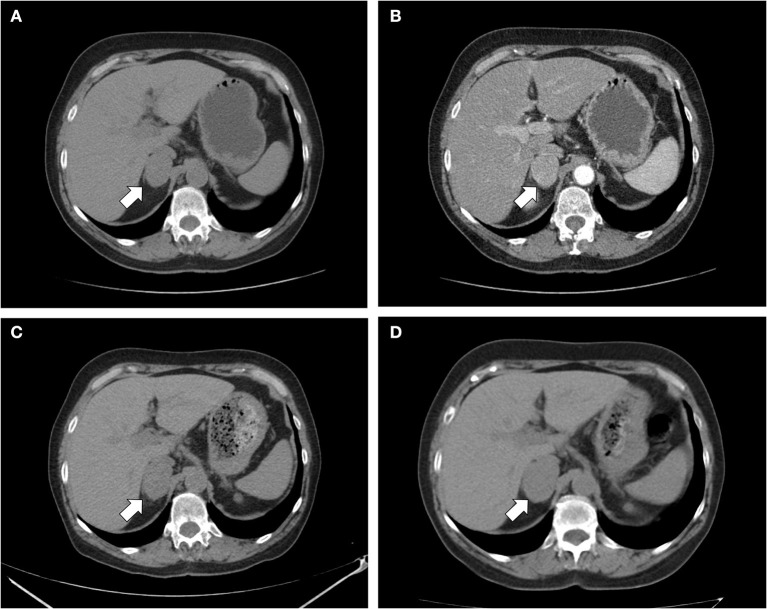
CT scan of the abdomen. **(A)** Unenhanced and **(B)** contrast-enhanced CT scan performed at diagnosis showed a homogeneous right adrenal mass of 32 × 40 mm with density of 35 HU and relative contrast washout of 21% (white arrow); **(C)** after 8 months, the CT scan showed a substantial stability of the adrenal lesion; **(D)** after 24 months, a slight dimensional growth of adrenal nodule (32 × 44 mm) and change in its density (39 HU) was found.

**Figure 2:**
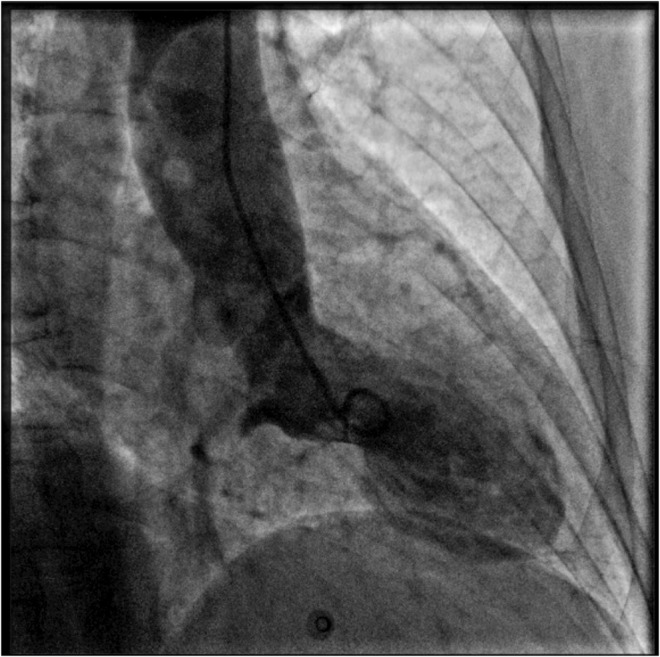
Coronary angiography and left ventriculography. Left ventriculogram during systole showing the typical apical akinesia and basal hyperkinesia of tako-tsubo cardiomyopathy.

**Figure 3:**
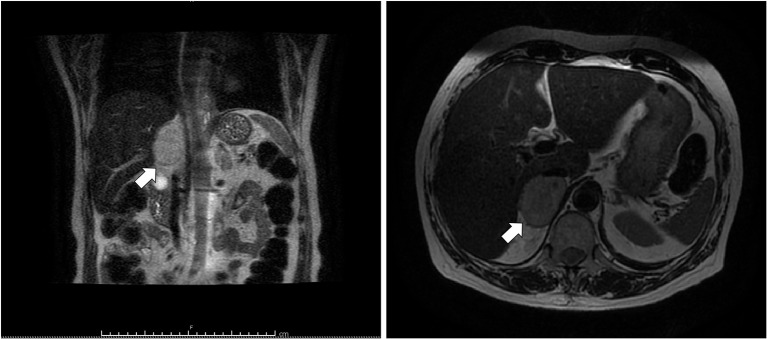
T2-weighted MRI of abdomen. Coronal (**left**) and axial (**right**) planes showing a hyperintense right adrenal mass (arrow) measuring 50 × 53 mm.

**Figure 4:**
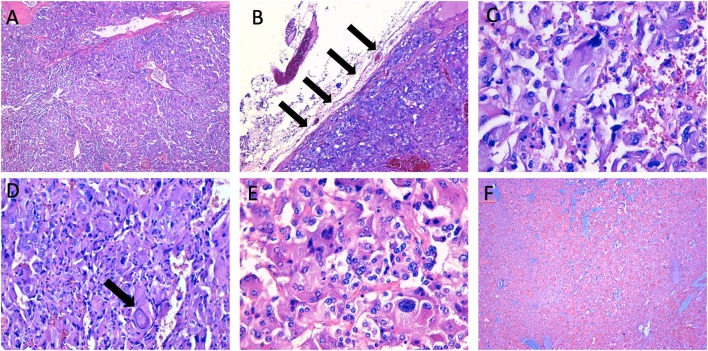
Histopathological characteristics of the right adrenal mass. **(A,B)** the neoplasm shows a high cellularity and a diffuse growth pattern, with partial adrenal capsule invasion (arrows) (haematoxylin-eosin, original magnification × 4, and × 10); **(C–E)** the tumoral cells show a high degree of hyperchromasia, with evidence of nuclear pleomorphism and large “pseudoinclusions” (arrow) (haematoxylin-eosin, original magnification × 20, and × 40); **(F)** immunohistochemical staining of synaptophysin (original magnification × 4).

A 69 years-old woman, with a history of papillary thyroid carcinoma, type 2 diabetes mellitus (T2DM) treated with metformin, and hypertension on treatment with angiotensin II receptor blockers (ARB), calcium antagonists, and β-blockers, was diagnosed in October 2015 as having a right-sided AI during a routine abdominal ultrasound exam. A contrast-enhanced computed tomography (CT) scan of the abdomen showed a homogeneous right adrenal mass of 32 × 40 mm with density of 35 HU and relative contrast washout of 21% (Figures 1A,B). Basal plasma cortisol, ACTH, and overnight 1 mg dexamethasone suppression test (DST) were normal. Similarly, 24 h urine cortisol, catecholamine, dopamine, and plasma vanilmandelic acid concentrations were within normal ranges. Plasma renin activity and aldosterone levels were not measured because of the concomitant therapy with ARB. The AI was then labeled as a non-functional benign adrenal adenoma. Clinical and biochemical characteristics were recorded yearly during the follow-up and shown in Table 1. The non-contrast CT-scan performed after 8 months from the diagnosis confirmed the substantial dimensional stability of the adrenal mass (Figure 1C). Given the indeterminate radiological features of the lesion, the same exam was repeated in October 2017, showing a slight dimensional growth of the nodule (32 × 44 mm) and change in its density (39 HU) (Figure 1D). Nevertheless, the patient was normotensive (110/80 mmHg) and T2DM was well controlled (HbA1c 51 mmol/mol), without clear signs or symptoms of adrenal hormones excess. In January 2018, the patient reported worsening of diabetes [average fasting plasma glucose (FPG): 185 mg/dL] and hypertension [mean blood pressure (BP) 150/95 mmHg] despite no treatment modification. For this reason, in May 2018 she was referred to our department for further investigations. On admission, she was completely asymptomatic, with a BP of 160/90 mmHg, a heart rate of 76 bpm, and FPG of 167 mg/dL. Her recent history was unremarkable, with no relevant signs or symptoms reported. During the second day of evaluation, she complained of palpitations, chest pain, diaphoresis, and profound asthenia. On examination, blood pressure and heart rate were high (210/115 mmHg; 115 bpm), while there was evidence of marked hyperglycemia (300 mg/dl) at the capillary blood glucose monitoring. Suspecting an acute coronary syndrome (ACS), she was sent to the emergency department, where the electrocardiogram (EKG) performed showed a diffuse ST elevation, with ST-depression in leads aVR and V1. The initial laboratory work-up showed: CK-MB 58.13 μg/L (normal <2.88), high-sensitivity Troponin I 1754 ng/L (normal <14), BNP 2623 pg/mL (normal <100), corroborating the diagnosis of acute myocardial infarction. Therefore, she underwent cardiac catheterization, revealing the absence of obstructing coronary artery disease. However, ventriculography showed left ventricular apical akinesia, basal hyperkinesia, and depressed systolic function, with an ejection fraction (LVEF) of 35% (Figure 2). Based on these findings, she was diagnosed as having TTS. In the following days, she developed type 1 respiratory failure, so she was admitted to the intensive care unit and treated with non-invasive ventilation. Intravenous urapidil, esmolol, and subcutaneous insulin were started for hypertension, tachycardia, and hyperglycemia, respectively, with a complete recovery after 72 h. Thus, she was transferred to our department for further investigations. Considering the patient's clinical history, a pheochromocytoma was suspected. A 24 h urine collection was performed and revealed high metanephrine (3795 μg/24 h, normal <350) and normetanephrine (2172 μg/24 h, normal <600) levels. A contrast-enhanced abdominal MRI was ordered, which displayed a right adrenal mass measuring 50 × 53 mm, with hyperintense T2-weighted images and a decrease in signal intensity of 12% (Figure 3). The patient was managed with doxazosin, atenolol, and massive hydration for 1 week, then she underwent laparoscopic right adrenalectomy, which was performed without any complication. Following the surgical procedure, all symptoms resolved. Blood pressure remained stable off all medications and glycemia was well-controlled with metformin. The histological examination showed a tumoral mass positive for chromogranin and synaptophysin immunostaining, with a Ki-67 index <1% and a PASS score of 7. Morphological and immunophenotypical findings were consistent with a diagnosis of pheochromocytoma (Figure 4). How suggested by international guidelines, several genes associated with pheochromocytoma were sequenced (SDHA, SDHB, SDHC, SDHD, SDHAF2, VHL, MAX, TMEM127, RET, EPAS1, FH, EGLN1, and KIF1Bβ), without any evidence of pathogenic mutations. The post-operative 24 h urine metanephrine levels were within normal reference values (metanephrine 20.9 μg/24 h; normetanephrine 268.3 μg/24 h) and BNP progressively returned to the normal value (210 pg/mL). She was discharged from hospital on 5th post-operative day, on therapy with aspirin and ace-inhibitor. We couldn't prescribe β-blocker because of the prolonged QTc at EKG. After 12 months of follow-up, the patient remained asymptomatic, with no echocardiographic or EKG stigmata of TTS.

**Table 1:** Clinical characteristics and laboratory findings at the diagnosis of adrenal incidentaloma (October, 2015), during the follow-up (June, 2016 and October, 2017), and after surgery.

	**AI diagnosis (Oct. 2015)**	**Jun. 2016**	**Oct. 2017**	**May 2018**	**After surgery**	**Ref. values**
**CLINICAL PARAMETERS**						
Weight (kg)	57	56	57	57	57	
Body mass index (kg/m^2^)	24.8	24	24.8	24.8	24.8	
Systolic blood pressure (mm Hg)	120	130	110	160	122	
Diastolic blood pressure (mm Hg)	75	80	80	90	66	
**BIOCHEMICAL PARAMETERS**						
Plasma cortisol at 8:00 am (μg/dL)	20.5	22	12.7		15.7	6–25
ACTH (pg/mL)	18.2	14.2	10.8		18	5–48
24 h urine cortisol (μg/24 h)	162.05	216	118			30–220
Plasma cortisol after 1 mg DST (μg/dL)	1.7					<1.8
24 h urine AD (μg/24 h)	12.9	11.7				2–25
24 h urine NA (μg/24h)	43.1	40.8				10–75
24 h urine dopamine (μg/24 h)	233.4	193.4				5–400
24 h urine VMA (mg/24 h)	10.5					1–13.6
24 h urine MN (μg/24 h)		188	160	3,795	20.9	<340
24 h urine NMN (μg/24 h)		250	212	2,172	268.3	<600

*AD, adrenaline; NA, noradrenaline; NM, metanephrine; NMN, normetanephrine; VMA, vanilmandelic acid*.

*Blood samples were taken in the morning (8 am) with the patient in the supine position*.

## Discussion

We reported the case of a pheochromocytoma presenting with TTS and originating from a non-functional adrenal adenoma diagnosed 3 years earlier. The prevalence of adrenal masses increases with age and is reported to be up to 10% in the elderly (1). In 80% of cases, AIs are non-functioning adenomas (1), and most of them remain hormonally inactive over time. However, a minority (0–11%) develop hyperfunction in their natural history (2). While cortisol is the most common hormone secreted, the onset of catecholamine secretion over time seems extremely rare (3), with only a few cases reported to date (4–8). In a study of Cho et al. (6) among 72 patients with non-functional AI monitored for a mean of 22.5 months, pheochromocytoma was diagnosed only in one patient. A recently published meta-analysis involving 2,745 patients with non-functional AIs showed that the risk for pheochromocytoma during a mean follow-up of 49.6 months was negligible (<0.1%), with only 3 patients reported in two studies (8). Determining the nature of an adrenal mass to decide the appropriate treatment is crucial and based on biochemical tests (1 mg dexamethasone suppression test; aldosterone-to-renin ratio; 24 h-urine and plasma metanephrines and normetanephrines; plasma 3-methoxytyramine) and imaging studies (CT, MRI, and FDG-PET/CT). The right timing of interval and duration of follow-up is still an open question. Current European guideline (1) suggests against repeated imaging and hormonal assessment in patients with small benign adrenal incidentalomas (<4 cm) and no evidence of hormonal secretion at the diagnosis, unless there is the development of signs or symptoms related to endocrine activity, like in the case here described. Indeed, in our patient, the adrenal lesion displayed a rapid change in its radiological features together with metabolic derangement over 12 months. Poor glucose tolerance and overt diabetes may be present in about 25–50% of patients with pheochromocytoma (9, 10). This manifestation is mediated by a multitude of mechanisms, including increased insulin resistance in peripheral tissues, impaired insulin secretion and glucose uptake (11). Catecholamine excess could worsen glycemic control in patients with pre-existing type 2 diabetes, while in rare cases, diabetes can be the only presenting feature of pheochromocytoma (12, 13). Tumor resection rapidly improves glucose homeostasis and, how observed in our patient, down-titration of insulin and antihyperglycemic agents is usually required. Overall, in individuals with adrenal adenomas and new-onset hyperglycemia or difficult-to-control diabetes, pheochromocytoma should be excluded. Other common signs and symptoms of catecholamine excess include tachycardia, hypertension, sweating, headache, and sense of anxiety. Diagnosis is based first on clinical suspicion and confirmed by elevated plasma free metanephrines or 24 h urine fractionated metanephrines (14). A 4-fold elevation above the upper limit value is associated with a near to 100% probability of the tumor presence (15). In our patient, 24 h urine metanephrines were 10-fold above the upper range limit, highly suggesting the presence of a pheochromocytoma. About one-fourth to one-third of all pheochromocytomas/PPGLs are familial and associated with various syndromes (16, 17). According to recent guidelines (18, 19), it has been recommended that all patients with apparently sporadic pheochromocytoma should be offered genetic testing. The most common germline mutations associated with familial pheochromocytoma are: REarranged during Transfection (RET) proto-oncogene, von Hippel-Lindau gene (VHL), neurofibromatosis type 1 gene (NF1), genes encoding four succinate dehydrogenase complex subunits (SDHx; i.e., SDHA, SDHB, SDHC, and SDHD genes), succinate dehydrogenase complex assembly factor 2 gene (SDHAF2), transmembrane protein 127 gene (TMEM 127), and myc-associated factor (MAX) (20, 21). Furthermore, cases of biochemically silent abdominal pheochromocytomas/PPGLs in individuals harboring the SDHB gene mutations have been reported (22). However, in our patient the genetic analysis resulted negative. Surgical resection of the tumor is the treatment of choice, but adequate pharmacological preparation before surgery is fundamental to avoid a potentially fatal intraoperative hypertensive crisis. Treatment is started with α-blockers, followed by β-blockers to control heart rate only when a complete α-blockage is achieved (23). Nearly 10% of all patients with pheochromocytoma could develop a catecholamine-induced cardiomyopathy, similar to that seen in TTS. Furthermore, as suggested by Y-Hassan et al. (24), when critically analyzed, numerous previously reported pheochromocytoma-associated cardiac dysfunctions not described as TTS have, as a matter of fact, hallmarks of TTS. The clinical presentation of TTS is often indistinguishable from that of an ACS, characterized by chest pain, dyspnea, or syncope, but in most of the cases there is no evidence of coronary artery disease (25, 26). In the last few years, an increasing number of pheochromocytoma-associated TTS has been reported (27). Patients with pheochromocytoma-associated TTS are generally younger and at higher risk of acute complications, compared to those with primary TTS. This could be related to the higher concentration of circulating catecholamines in patients with actively secreting pheochromocytoma/paraganglioma (28). Direct toxic effect of catecholamine on the myocardium and indirect mechanisms, like peripheral vasoconstriction, tachycardia, and coronary vasospasm, have been suggested to contribute to the cardiac dysfunction observed in TTS (29). As catecholamine levels are usually elevated in TTS (29), β-blockers seem to be reasonable in the acute phase, until full recovery of LVEF, although clear data from randomized trials are lacking. However, β-blockers should be used cautiously in patients at risk for arrhythmias (e.g., bradycardia; prolonged QTc), and as long as an underlying pheochromocytoma has been ruled out. Indeed, in patients harboring an actively secreting pheochromocytoma/PPGL, the administration of β-blockers before reaching a complete α-blockage could lead to a massive unopposed α-stimulation, potentially precipitating a severe hypertensive crisis. Historically, Mayo Clinic criteria (30) have been proposed to help clinicians to diagnose TTS but, based on current knowledge, the HFA-ESC task force has recently developed new international tako-tsubo Diagnostic Criteria (InterTAK Diagnostic Criteria) (26). In these guidelines, for the first time, pheochromocytoma was included among secondary causes of TTS. Since the reported prevalence of pheochromocytoma/PPGL is significantly higher in patients with TTS (7.5%) compared to other highly selected populations (0.2–0.6% of all patients with hypertension), screening for a catecholamine-secreting tumor should be considered in all patients presenting with TTS (31–35).

## Conclusions

Although it is unlikely that a non-functional AI may develop clinically overt hormone excess during its natural history, we described a rare case of non-secretory adrenal adenoma developing catecholamine secretion after several years of clinical, biochemical, and radiological follow-up. In selected cases, long-term follow-up may be necessary for early detection of these rare, unpredictable tumors, paying particular attention to the onset of new symptoms or worsening of comorbidities. In our patient, hormonal activity presented with a dramatic symptomatology and life-threatening TTS caused by the catecholamine surge. Based on the recently published consensus statement and increasingly reported cases, a diagnosis of pheochromocytoma should always be taken into account when echocardiography or ventriculography reveals the typical TTS appearance.

## Data Availability Statement

All datasets generated for this study are included in the article/supplementary files.

## Ethics Statement

This study represents a case report. All the procedures in this case were conducted according to guidelines and according to clinical practice. All procedures performed in studies involving human participants were in accordance with the ethical standards of the Ethics committee of University Hospital of Pisa and with the 1964 Helsinki declaration and its later amendments or comparable ethical standards. A written informed consent was obtained from the patient for the publication of this case report and any potentially-identifying images/information.

## Author Contributions

PF and MT wrote the manuscript. PF and FO followed the patient. All authors contributed to manuscript revision and approved the submitted version.

### Conflict of Interest

The authors declare that the research was conducted in the absence of any commercial or financial relationships that could be construed as a potential conflict of interest.

## Abbreviations

ACS: acute coronary syndrome
AI: adrenal incidentaloma
BNP: brain natriuretic peptide
BP: blood pressure
CK-MB: creatine kinase myocardial band
CT: computed tomography
EKG: electrocardiogram
FPG: fasting plasma glucose
LVEF: left-ventricular ejection fraction
MRI: magnetic resonance imaging
TTS: tako-tsubo syndrome.
